# Hepatokines, bile acids and ketone bodies are novel Hormones regulating energy homeostasis

**DOI:** 10.3389/fendo.2023.1154561

**Published:** 2023-05-19

**Authors:** Gabriella Garruti, Jacek Baj, Angelo Cignarelli, Sebastio Perrini, Francesco Giorgino

**Affiliations:** ^1^Unit of Internal Medicine, Endocrinology, Andrology and Metabolic Diseases, Department of Precision and Regenerative Medicine, University of Bari Aldo Moro, Bari, Italy; ^2^Department of Anatomy, Medical University of Lublin, Lublin, Poland

**Keywords:** bile acids, fasting, GPBAR1, hepatokines, ketogenic diet

## Abstract

Current views show that an impaired balance partly explains the fat accumulation leading to obesity. Fetal malnutrition and early exposure to endocrine-disrupting compounds also contribute to obesity and impaired insulin secretion and/or sensitivity. The liver plays a major role in systemic glucose homeostasis through hepatokines secreted by hepatocytes. Hepatokines influence metabolism through autocrine, paracrine, and endocrine signaling and mediate the crosstalk between the liver, non-hepatic target tissues, and the brain. The liver also synthetizes bile acids (BAs) from cholesterol and secretes them into the bile. After food consumption, BAs mediate the digestion and absorption of fat-soluble vitamins and lipids in the duodenum. In recent studies, BAs act not simply as fat emulsifiers but represent endocrine molecules regulating key metabolic pathways. The liver is also the main site of the production of ketone bodies (KBs). In prolonged fasting, the brain utilizes KBs as an alternative to CHO. In the last few years, the ketogenic diet (KD) became a promising dietary intervention. Studies on subjects undergoing KD show that KBs are important mediators of inflammation and oxidative stress. The present review will focus on the role played by hepatokines, BAs, and KBs in obesity, and diabetes prevention and management and analyze the positive effects of BAs, KD, and hepatokine receptor analogs, which might justify their use as new therapeutic approaches for metabolic and aging-related diseases.

## 1 Introduction

In humans and other large mammals, energy homeostasis depends on several complex pathways that control the balance between energy intake and energy expenditure. For a long time, obesity was considered the effect of an imbalance between energy expenditure and intake, but actual data support a much more complex picture. The “dogma” of impaired balance explains only part of the mechanisms responsible for body fat excess. Growing evidence indicates that fetal malnutrition (1, 2), early exposure to endocrine-disrupting chemicals (EDC) (3–6), and early exposure to EDC contribute to obesity, visceral fat accumulation (3, 7–11), and insulin resistance (10, 12, 13).

Involved mechanisms include gene-environment crosstalk (2, 14, 15) and epigenetic changes of DNA methylation (16), histone acetylation/deacetylation, and non-coding mRNA (16). The early and/or prolonged exposure to an “obesogenic” environment, together with environmental factors (2, 17) activates pathways that lead to inflammation and immune system dysfunction involved in chronic low-grade inflammation and insulin resistance (18, 19). Insulin resistance occurs when insulin binding to its receptor is not followed by (1) adequate glucose uptake into target tissues such as the skeletal muscle and adipose tissues (2), suppression of glucose production by the hepatocytes, and/or (3) decreasing of adipose tissue lipolysis (20).

In the present review, we will focus on possible roles played by the liver as a source of molecules playing endocrine function and regulating energy homeostasis.

## 2 The role of the liver in hepatokines, ketone bodies, bile acids synthesis, and metabolism

The liver plays a major role in systemic glucose homeostasis. It senses nutrient availability and increases or decreases glucose production and glycogen storage in the transition from fasting to the post-prandial phase. After an overnight fast, glucose supply to the brain and muscles is ensured by increased hepatic glycogenolysis and gluconeogenesis (21, 22). By contrast, in the post-prandial phase, hepatic glucose production is reduced and hepatic glucose uptake is increased (enhanced glycogen-synthesis and the novo lipogenesis) because circulating levels of glucose are sufficient for the energy needs of non-hepatic organs (23, 24).

**Figure 1:**
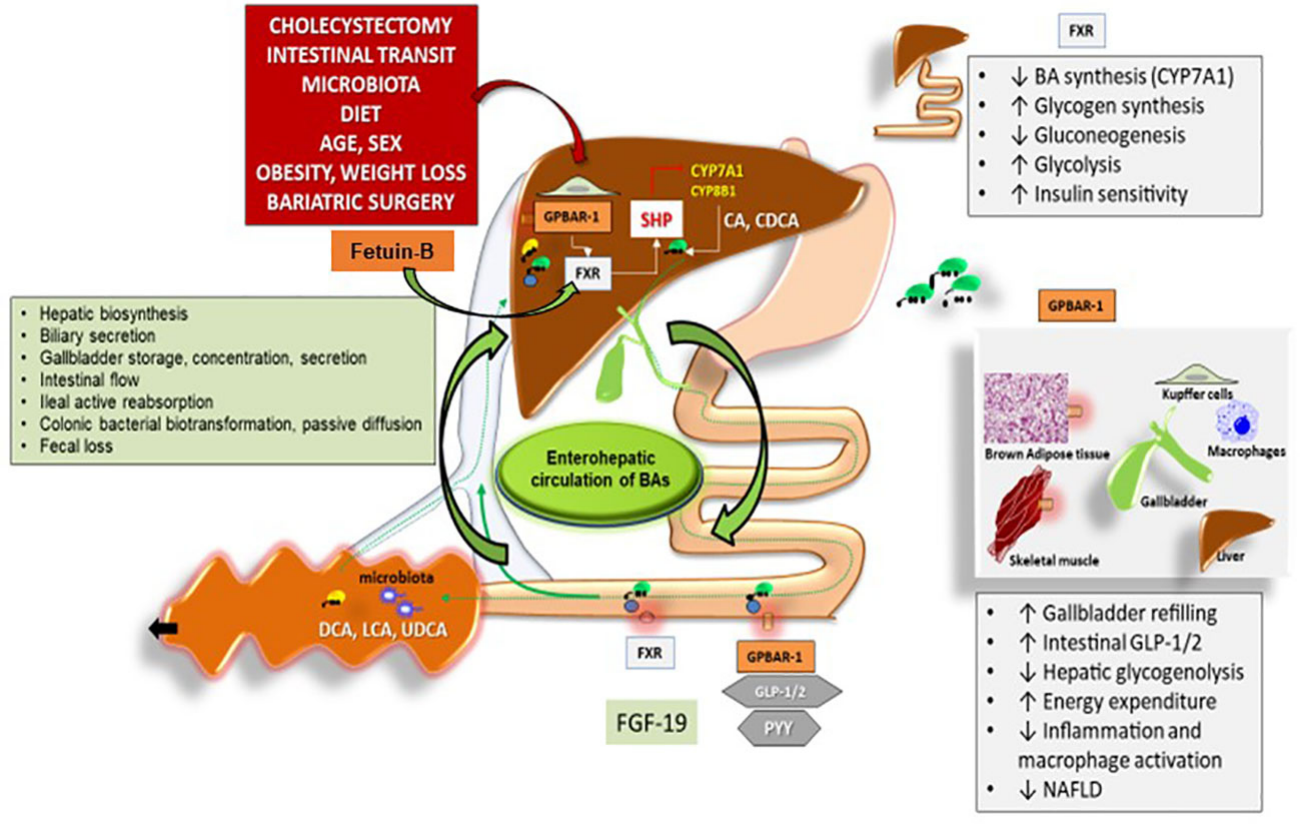
Interaction between hepatokines and BAs in nutrient and energy homeostasis. Legend for Figure 1 Overall events involved in the function of bile acids (BAs) acting as signaling molecules and receptor ligands of farnesoid X receptor (FXR) and the G protein–coupled receptor 1 (GPBAR–1). In the left box, the steps involved in BA biosynthesis, secretion, storage, intestinal flow, absorption, colonic biotransformation and fecal loss are listed, as part of the enterohepatic circulation of BAs. The location of FXR and GPBAR–1 (TGR5) are shown in the liver, intestine, and several other tissues with respect to the control of BAs on: 1. hepatic biosynthesis of primary BAs *via* the rate–limiting enzyme 7a–hydroxylase (CYP7A1) and CYP8B1 controlled negatively by the small heterodimer partner (SHP): cholic acid (CA) and chenodeoxycholic acid (CDCA), 2. intestinal release of FXR–mediated secretion of the fibroblast growth factor 19 (FGF19, known as FGF15 in mice), which circulates to the liver and reduces the expression of CYP7A1 to inhibit hepatic BA synthesis through FXR, 3. Intestinal GPBAR–1–mediated release of peptide YY (PYY), glucagon–like peptide 1 (GLP–1) and glucagon–like peptide 2(GLP–2). Recent studies show that Fetuin–B is playing additional roles through its interaction with Farnesoid X Receptor (FXR). In studies utilizing microarray technology Fetuin–B acts as a FXR agonist–regulated gene. Several metabolic functions (boxes on the right). Adapted from 25 modified by Garruti et al. for Front Endocrinol.

**Figure 2:**
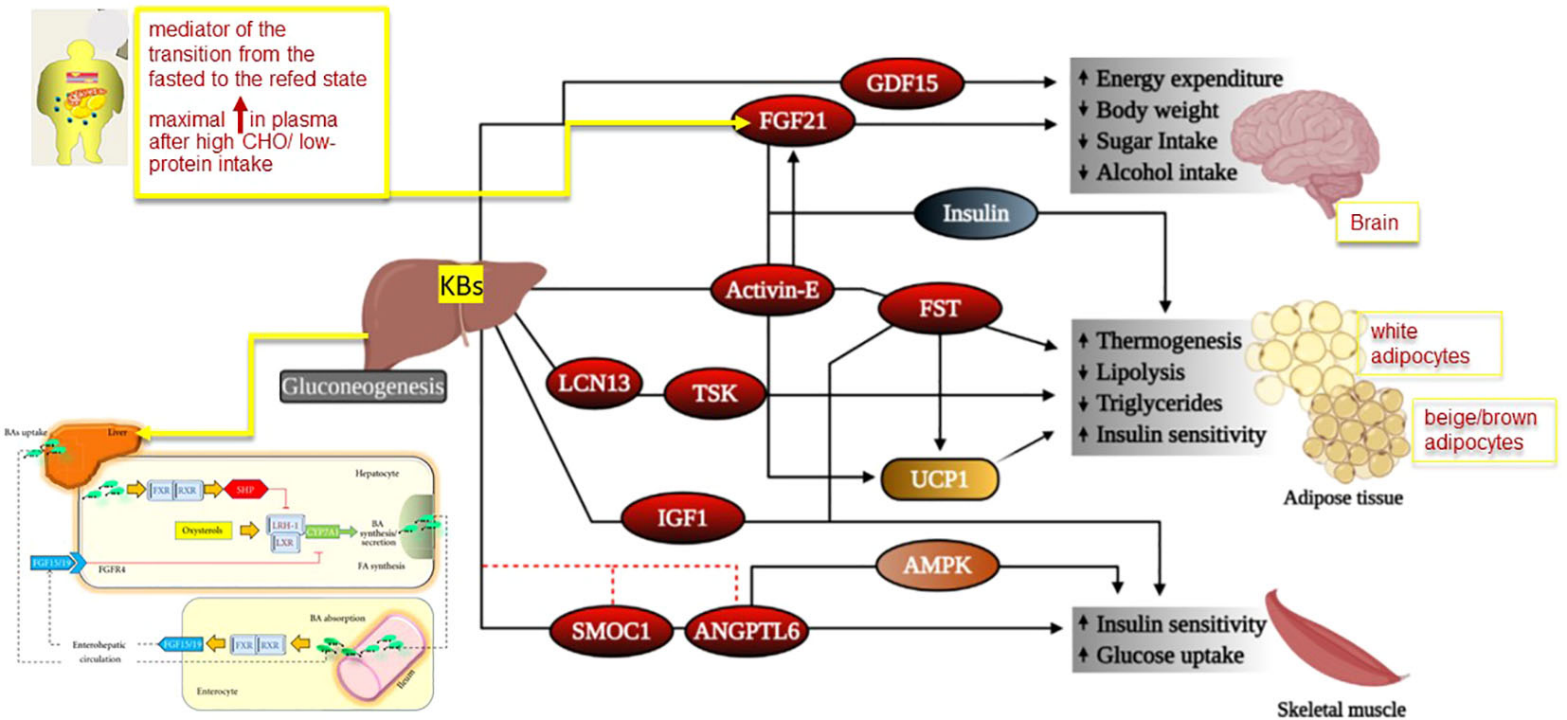
Mechanism for physiological effects of hepatokines in target tissues. Legend for Figure 2 Some hepatokines signal the brain to modulate body weight homeostasis and food intake, while others act on adipose tissues and muscle to regulate lipid and glucose homeostasis. However, liver is also the main site of production of bile acids (BAs). In the figure we depicted the potential molecular mechanisms of crosstalk between nuclear receptors LXR and FXR–SHP–LRH–1 regulatory cascade in the liver and intestine. Bile acids are the natural ligands for FXR, which regulates transcription by binding as a heterodimer with RXRs. This step results in increased SHP expression. SHP in turn inhibits LRH–1, preventing the activation of target genes that participate in bile acid and fatty acid synthesis. In the absence of bile acids, LRH–1 acts together with LXR to stimulate bile acid synthesis. The important pathways in the intestine that contribute to modulation of bile acid synthesis are also depicted. There is a bile–acid–mediated activation of intestinal FXR and, as a result, the release of FGF15 in the small intestine. The secreted FGF15 by the intestine circulates to the liver, likely through the portal circulation or lymph flow and induces the activation of FGFR4 in the liver. The FGF15/FGFR4 pathway synergizes with SHP in vivo to repress CYP7A1 expression. Abbreviations: AMPK, adenosine monophosphate–activated protein kinase, ANGPTL6, angiopoietin–like 6,BAs: bile acids, FGF: fibroblast growth factor, FGFR4: FGF receptor 4, FGF21, fibroblast growth factor 21, FST, follistatin, FXR: farnesoid X receptor, GDF15, growth differentiation factor 15, IGF1, insulin–like growth factor 1, LCN13, lipocalin 13, LRH–1: liver receptor homologue–1, LXR: liver X receptor, RXR: retinoid X receptors, SHP: short heterodimer partner, SMOC1, SPARC–related modular calcium–binding protein–1, TSK, Tsukushi, UCP1, uncoupling protein 1 Adapted from Reference 26 and 27 modified by Garruti et al. for Front Endocrinol.

In humans and other mammalian species, the liver is also the main site of the production of bile acids (BAs), ketone bodies (KB), and lipids (**Figures 1**, **2**). The liver supplies KB derived by lipids oxidation and very low-density lipoproteins (VLDL) to target tissues.

During short-term fasting, mammalian species produce and use KB as a surrogate fuel to compensate for the decrease in carbohydrate (CHO) availability and the increase in fatty acid availability (28, 29). In prolonged fasting, the brain utilizes KB as an alternative to CHO. In prolonged energy deprivation, either eukaryotes or archea and bacteria uses KB (28).

Hepatokines are proteins secreted by the hepatocytes. They influence metabolism through autocrine, paracrine, and endocrine signaling and are the main mediators in the crosstalk between the liver, non-hepatic target tissues, and the brain. Hepatocytes secrete more than 500 hepatokines (30).

Many hepatokines have been linked to the induction of metabolic dysfunction. Altogether, hepatokines represent the hepatocyte secretome, which is consistently modified in pathological conditions. In liver steatosis, increased intrahepatic depots of triglycerides are associated with changes in transcription and endoplasmic reticulum (ER) folding and the transport of some hepatokines and are ultimately causing the increase in secretion of some of them and the decrease in secretion of some others. This imbalance in hepatokines levels is associated with insulin resistance, glucose intolerance, ectopic lipid accumulation, inflammation, and impaired insulin secretion. Robust data indicate that Fetuin A, Fetuin B, Retinol-binding protein 4 (RBP4), and Selenoprotein P are key players in metabolic dysfunction (31, 32). The present review will focus on some of the hepatokines playing a role in obesity, diabetes, and non-alcoholic fatty liver disease (NAFLD) not only in animal models but also in humans.

## 3 Hepatokines

In this section, we will consider some of the hepatokines already studied in humans that are related to obesity, insulin resistance, and/or diabetes, and NAFLD. Angiopoietin-like proteins (ANGPTL) are eight subtypes of glycoproteins secreted by the liver. They share with the angiopoietin proteins the same C-terminal fibrinogen-like domain and the N-terminal coiled-coil domain (33). ANGPTL3 is one of the most studied ANGPTLs in humans. The liver is the only site of ANGPTL3 synthesis. Cohorts of subjects with loss of function alleles for ANGPTL3 show reduced circulating levels of both triglycerides (TGL) and cholesterol (LDL and HDL) (26, 34). Similar findings exist in knockout mice for ANGPTL3 that exhibit reduced blood levels of TGL and free fatty acids because of the increased activity of LPL (35, 36). The interaction between ANGPTL3 and LPL induces the dissociations of LPL dimers to monomers. The transformation of the active to the inactive LPL accounts for the enhanced deposition of FFA derived by lipoproteins into white adipocytes (37). Interestingly, the liver X receptor (LXR) upregulates ANGPTL3. By contrast insulin, peroxisome proliferator-activated receptor (PPAR) beta and leptin downregulate it. Some recent studies indicate that thyroid hormones and statins are also negatively regulating ANGPTL3 and might explain its effects on lipid and CHO metabolism (38, 39).

Evinacumab is a synthetic ANGPTL3 agonist. In recent studies, combining Evinacumab with either statins or PCSK inhibitors, rodents showed a more significant improvement in hyperlipidemia compared with statins alone (40).

ANGPTL3 is not only regulating lipid patterns but is also involved in glucose homeostasis. In insulin-resistant subjects, ANGPTL3 levels directly correlated with HOMA-IR as well as with glucose and insulin circulating levels (41).

ANGPTL6 is expressed in different tissues at very low levels but hepatocytes are the major source of its secretion. In animal models, overexpression of ANGPTL6 is associated with increased energy expenditure, protection of hepatic steatosis, resistance to high-fat diet-induced overweight, and increased insulin sensitivity compared with controls. By contrast, knockout mice for ANGPTL6 show an increased incidence of obesity, insulin resistance, and increased fat content in peripheral organs (especially the liver and skeletal muscle) (42). Another effect of ANGPTL6 on glucose metabolism is represented by the reduced expression in glucose-6 phosphatase and the following decrease in gluconeogenesis (43, 44). Most recent data in humans indicate that subjects with obesity and/or type 2 diabetes have higher blood levels of ANGPTL6 compared with controls (45).

In both humans and mice, fasting–induced adipose factor (FIAF) is another very important hepatokine. It is mainly expressed in adipocytes and hepatocytes, but it might also be detected in cardiomyocytes and skeletal muscle myocytes (46, 47). It is also known as ANGPTL4. Fasting was the first condition known to induce a robust increase in hepatic levels of mRNA for FIAF while refeeding was able to suppress FIAF expression (48). It stimulates adipose tissue lipolysis, inhibits LPL activity, and blocks the clearance of triglyceride–rich lipoproteins. These mechanisms account for the increase in plasma FFA and triglycerides (49, 50). During food deprivation, there is a decrease in LPL concentrations in adipose tissue capillaries and a prevailing triglyceride uptake in oxidative target tissues (myocytes of skeletal muscle) (51). Very recently, research has demonstrated that FIAF expression increases during exercise, and triglyceride uptake is shifted from the adipose tissue to the muscle with the same mechanism activated during fasting (52). Contrasting results exist on FIAF circulating levels in obesity and type 2 diabetes (53). In knockout mice for FIAF, a high–fat diet is associated with a higher increase in visceral fat compared with controls (54). The phenotype of mice with the overexpression of FIAF is interesting and characterized by liver steatosis but there is also a better insulin sensitivity in the liver and other major target tissues (55). FIAF is not only playing a role in energy homeostasis but is involved in angiogenesis and cancer cell infiltration.

Fetuins are abundant fetal serum α–globulins that belong to the cystatin family of cysteine protease inhibitors (56). The α2–Heremans and Schmid glycoprotein (AHSG) are also known as Fetuin–A. Human Fetuin–B (382 amino acids) shares 22% sequence similarity with Fetuin–A. Fetuin–A is coded by the AHSG gene and Fetuin–B by the Fetub gene. Fetuin–A was first described because of its role in osteogenesis and the inhibition of vascular calcification and bone reabsorption (57, 58). Unexpectedly, Fetuin–A might also negatively regulate the activity of the insulin–receptor tyrosine kinase in the liver, adipose tissue, and skeletal muscles (59–61). Fetuin–B is also able to inhibit calcium phosphate precipitation, but since in humans its circulating levels are low, its role in osteogenesis and bone reabsorption seems limited. In wild–type mice, Fetuin–B inhibits the activity of ovastacin, a metalloproteinase, which is responsible for the hardening of the zona pellucida. By contrast, female Fetuin–B deficient mice are infertile because of the enhanced activity of ovastacin (62).

Recent studies show that Fetuin–B plays an additional role through its interaction with Farnesoid X Receptor (FXR or NR1H4). In studies utilizing microarray technology, Fetuin–B acts as an FXR agonist–regulated gene (**Figure 1**). FXR belongs to a nuclear receptor family, is mainly activated by bile acids (BAs), and is abundantly expressed in the adrenal gland, intestine, liver, and kidney (63, 64). Recent data support the role of Fetuin–B in insulin resistance. Adult subjects with liver steatosis and/or type 2 diabetes have increased levels of Fetuin–B. In cell cultures of hepatocytes and myocytes, Fetuin–B impairs insulin activity. In support of the insulin–resistant role of Fetuin–B, there are also animal studies showing that mice treated with Fetuin–B develop impaired glucose tolerance (31).

Recent studies demonstrate that Fetuin–A and adiponectin have opposite effects on insulin sensitivity. Adiponectin is a cytokine playing a role in chronic low–grade inflammation and positively correlates with insulin sensitivity (65). By contrast, Fetuin–A positively correlates with insulin resistance. Fetuin–A inhibits adiponectin and vice versa, adiponectin is a negative regulator of Fetuin–A through the AMPK signaling cascade. In subjects diagnosed with metabolic syndrome, the increased circulating levels of Fetuin–A might be due to the decreased expression of adiponectin (66). Studies on type 2 diabetic patients show that Fetuin–A concentrations are decreased by pioglitazone, as well as three months of reduced caloric intake causing a reduction in intra–abdominal body fat, arterial blood pressure levels, and fasting glycemia, together with an improvement in lipid patterns (67, 68).

Hepatocytes are the major site of synthesis and secretion of another hepatokine and fibroblast growth factor 21 (FGF21). FGF21 is defined as a fasting hormone, and it is a key mediator of lipid metabolism during fasting ketosis (69) (**Figure 2**). It is regulated by the nuclear receptor peroxisome proliferator–activated receptor alpha (PPARα). In high–fat–fed Rhesus macaque monkeys, FGF21 administration reduced body weight without changing energy intake (70). In mouse models of insulin–resistance treatment, FGF21 increased energy expenditure, reduced plasma circulating levels of glucose, improved liver steatosis, and improved both leptin and insulin sensitivity (71, 72). FGF21 binds the co–receptor b–klotho (KLB) and facilitates the formation of the FGF receptor 1/KLB complex, which is involved in the phosphorylation of ER1/2 (73). Recently, research demonstrated that FGF21 controls the expression of some genes involved in cellular aging and energy homeostasis like acetyl–Coa carboxylase (ACC1) and adipose triglyceride lipase. AMPK is the downstream protein of FGF21 and promotes lipolysis. However, FGF21 also regulates the expression of ACC1 and SREBP1c, thus inhibiting lipid synthesis. The anti–aging effects of FGF21 might also account for AMPK activation which plays a key role in the regulation of mTORC1 gene and NF–kB genes involved in autophagy and inflammation, respectively (74). FGF21 is considered a mediator of the transition from the fasted to the refed state because it stimulates insulin–mediated glucose uptake (32). In mice and humans, the maximal increase in plasma levels of FGF21 occurs after a high intake of simple CHO combined with low–protein intake (75). However, Schumann and co–workers demonstrated that FGF21 synthesis also increases after alcohol consumption (76). In subjects with obesity and/or type 2 diabetes and NAFLD, FGF21 circulating levels are higher than those measured in healthy age–matched subjects. The possibility exists that these metabolic conditions are characterized by FGF21–resistance and increased levels of FGF21 might be a compensatory mechanism (74). Some analogs of FGF21 already exist. Their administration to obese subjects with type 2 diabetes is associated with weight loss, a decrease in fasting insulin levels, and improvement in triglyceride circulating levels (74, 77). FGF21 analogs or FGF21 receptor agonists might represent an alternative treatment for NAFLD, obesity–associated type 2 diabetes, and other aging–associated metabolic diseases. FGF21 also plays an important role in the brain, involving potential effects in metabolic regulation, neuroprotection, and cognition (78).

Lipasin is an angiopoietin–like protein (ANGPTL8 or betatrophin). In humans and mice, it is expressed in the liver and adipose tissue. Food intake–mediated insulin secretion, FFA, and thyroid hormones regulate the levels of the expression of lipasin (79). Lipasin plays an anorectic effect by regulating the activity of NPY in the dorso–medial hypothalamus (80). It seems to negatively regulate glucose and lipid metabolism. Lipasin forms a complex with ANGPTL3 which robustly inhibits LPL. When ANGPTL4 is bounded to lipasin, ANGPTL4 loses its ability to inactivate LPL.

Selenoproteins are glycoproteins that are also secreted by the hepatocyte. Selenium is essential in balanced nutrition in humans and animal models. It was originally studied because of its role in thyroid diseases. Very recently, it was also found to play a role in fat and CHO metabolism. In the blood, selenium is bound to cysteine (selenocysteine) or proteins (selenoproteins). Selenoprotein P carries and donates selenium to peripheral target organs (81). Circulating levels of selenoprotein are positively regulated by selenium and glucose intake. Adiponectin and insulin reduce the expression of selenoprotein P (82, 83). Animal models helped to understand the role of selenoprotein in insulin resistance. The treatment of wild–type mice with selenoprotein P was associated with the appearance of impaired insulin sensitivity and impaired glucose tolerance. This treatment improved both insulin sensitivity and glucose tolerance in knockout mice for selenoprotein P (82). In patients diagnosed with type 2 diabetes, obesity or NAFLD serum levels of selenoproteins are higher compared with those measured in healthy subjects (83, 84). Prestigious studies show increased circulating levels of selenoprotein P associated with hypertriglyceridemia and insulin resistance (84).

## 4 Ketone bodies and ketogenic regimens

In mammals, several physiological conditions are characterized by the oxidation of ketone bodies to produce energy. In humans and other mammalian species, the liver is the major site of production of ketone bodies (KB). The liver diverts KB derived by lipids oxidation and very low–density lipoproteins (VLDL) to target tissues.

During short–term fasting, mammalian species might utilize KB as a surrogate supply to compensate for decreased CHO availability and increased fatty acid availability (28, 29). In prolonged fasting, the brain also utilizes KB as an alternative to CHO.

In healthy humans, the daily hepatic production of KB is around 300 grams. Prolonged starvation induces robust ketone body production and utilization, but KB production is also followed by intense exercise. In adults, normal circulating levels of KB range from 100 to 250 µM. After prolonged exercise or 24h food deprivation, KB increases 10 times (28), and in pathological conditions such as ketoacidosis, they might increase 200 times (28).

Studies in subjects undergoing ketogenic diets show that KBs are important mediators of inflammation and oxidative stress and might influence the post–translation re–arrangement of proteins (85, 86).

In the last 40 years, the ketogenic diet (KD) became a promising dietary intervention in some pathological conditions. The main outcome of this dietary strategy is to reach nutritional ketosis, but KD is usually assimilated to very low–carbohydrate diets where carbohydrates (CHO) are ranging from 5 to 10% of a 24h caloric intake independent of the total caloric intake (87, 88). It is not possible to establish a general cut–off level for CHO and kilocalories (Kcal) under which all individuals enter ketosis because this cut–off is subjective (89). In a high–fat ketogenic diet (HFKD), the daily CHO intake represents less than 50 g, but there are no limits for fat and caloric intake. HFKD was originally studied as a non–pharmacologic therapy in children and adolescents diagnosed with refractory epilepsy. Unfortunately, fatal cardiac arrhythmias (prolonged QT interval) and cardiomyopathy occurred in a few epileptic children treated with HFKD, supplying less than 800 kcal/day (VLCD) (90). More than 40 years ago, some retrospective studies concerning adult obese subjects assuming liquid–protein–modified fast diets poor in selenium reported some cases of sudden death associated with prolonged QT interval (91). Because of these sudden deaths, the European Food Safety Authority published precise guidelines stating that VLCKD should supply adequate selenium intake not less than 30 to 50 g/day of CHO but not more than 15 to 30% of fats for total kcal/day (92). In 2019, in a prospective cohort study, KD was associated with an increased risk of arrhythmia (atrial fibrillation). However, this KD was a high–fat/low–CHO diet (93).

A ketogenic diet with low CHO and high–fat content mimics the metabolic state of starvation. Recent trials considered a ketogenic diet as a therapeutic strategy in children with intractable epilepsy (94). Even if this therapeutic approach was successful in reducing epileptic crisis, it was also associated with thyroid dysfunction (95).

Most recent formulas of KD are represented by very low–calorie KD (VLCKD) supplying 400 to 800 kcal/day, but they are always supplying adequate selenium supplementation (96, 97). They are actually promising weight loss strategies for overweight/obese people. The majority of VLCKD is also known as protein–sparing modified fasting because they contain increased protein intake, mainly vegetable protein intake, which counterbalances the reduced CHO content (98).

The most intriguing observational studies analyzed the effects of ketone bodies on the myocardium. In mice, preliminary studies demonstrated that failing hearts consumed higher amounts of ketone bodies than healthy hearts (99). The same result was confirmed in humans (100–102). In experimental models of ischemia following reperfusion, KBs seem to be protective (103). Circulating levels of KBs were increased in subjects with heart failure, but the mechanisms involved are still unknown (104, 105).

Recently, the current treatment of type 2 diabetes with inhibitors of tubular sodium/glucose co–transporter–2 (GLT2i) highlighted other important roles played by KBs. In both mice and humans, the inhibition of sodium/glucose co–transporter–2 at the level of the proximal tubules of the kidney was associated with an increase in liver ketogenesis, which accounted for the increased blood levels of KB (106–108). The treatment of type 2 diabetes with the new GLT2i is associated with a reduction in cardiovascular mortality and risk of hospitalization for heart failure. Therefore, GLT2i–induced ketosis might represent one of the beneficial effects of these drugs, together with reduced plasma volume due to osmotic diuresis, decreased body weight, reduced arterial blood pressure levels, and improvement in sympathetic nervous system activity, glycemia, and hyperuricemia (109–111).

## 5 Bile acids are nutrient signaling and thermogenic hormones

Bile acids (BAs) are the major lipid components of bile. The liver synthetizes BAs from cholesterol and secretes them into bile (**Figure 1**). The gallbladder (GB) represents the physiologic storage compartment of BAs. After food consumption, the entero–hormone cholecystokinin (CCK) stimulates GB to release bile into the duodenum where the BAs–mediated digestion and absorption of fat–soluble vitamins and lipids occurs (112, 113). Cyclic changes in serum BAs concentrations accompany the fasting to fed transition. In healthy subjects, fasting serum BAs concentrations are 0.2 to 0.7 µM, and after meals, they reach 4 to 5 µM (114–116). One part of the BA is re–absorbed in the ileum and returned to the liver through the portal vein. The recycling of BAs is a negative feedback regulatory mechanism that inhibits further hepatic BAs synthesis. Another part of BA escapes from intestinal reabsorption, enters the colon, and gives rise to secondary BA by the resident gut microbiota (25).

Recent studies unequivocally pointed out that bile is not simply a fat emulsifier. BAs indeed represent signaling molecules regulating different metabolic pathways involving the nuclear receptors farnesoid X receptor (FXR), pregnane X receptor (PXR), vitamin D receptor (VDR), G–protein coupled receptors (TGR5 or GPBAR1), c–Jun N–terminal kinase (JNK), and extracellular signal–regulated kinase (ERK). Through these interactions, BAs are able to affect energy, glucose, lipid, and lipoprotein metabolism (117).

FXR is mainly activated by BAs and is abundantly expressed in the adrenal gland, intestine, liver, and kidney (63, 64).

FXR is mainly involved in cholesterol and triglyceride metabolism. However, some reports emphasized FXR activity in glucose homeostasis (118). Knockout mice for the FXR gene show impaired insulin sensitivity (118, 119). FXR negatively regulates lipogenesis and glucose production in the liver (120, 121) (**Figure 1**). Bile acids (BAs) are not only the natural ligand of FXR but also the major ligand for GPBAR–1 (TGR5). FXR is usually interacting with DNA by forming a heterodimer with inverted repeat–1 (IR1) elements like Retinoid X receptor (RXR). FXR is the principal BA sensor in the liver and ileum (122, 123). FXR activation increases the expression of fibroblast growth factor 19 (FGF19) in the human intestine and that of fibroblast growth factor 15 (FGF15) in mice intestines. When the circulating FGF19 enters the portal vein and reaches the liver, it induces a reduction in the expression of hepatic cholesterol 7D–hydroxylase in BAs’ additional synthesis. (124).

BAs are also the natural ligands of GPBAR–1 largely expressed in brown adipocytes, Kupffer cells, entero–endocrine cells of the intestine, macrophages, monocytes, and skeletal muscle. The effects of the binding of BAs with GPBAR1 are site–specific (**Figure 1**). In ileal L–cells, the binding of BAs to GPBAR–1 increases the secretion of PYY, Glucagon–like peptide (GLP)–1, and GLP2 with anorexigenic effect (111). In macrophages and Kupffer cells, GPBAR–1 activation blocks LPS–induced cytokine production. In skeletal muscle and brown adipocytes, GPBAR–1 signaling results in paracrine local activation of type II iodothyronine deiodinase (DIO2) which catalyzes the transformation of thyroxine (T4) to the active triiodothyronine (T3) responsible for increased energy expenditure (125). Recent research has demonstrated additional roles of BAs beyond their digestive function (114). By considering these additional effects, BAs are unequivocal mediators of glucose/insulin homeostasis and might explain some positive effects of bariatric surgery which appear independently of body fat loss (65, 113, 125, 126). Bariatric surgery represents an interesting model to re–evaluate the function of BAs in the pathophysiology of weight loss following surgical procedures in obesity (127).

Several papers demonstrated that plasma BA levels increase after bariatric surgery and might induce GLP–1 secretion in the intestine (128). Nonetheless, BAs were demonstrated to activate thyroid hormones (126, 129–133). This view is mainly supported by animal studies. GPBAR–1 knockout mice fed with a high–fat diet show a higher amount of body fat mass compared with wild–type mice. Interestingly, by increasing GPBAR–1 expression with the GPBAR–1 agonist INT–777, HFD–induced obesity is blunted (128, 134, 135).

Unexpectedly, at the level of brown adipose tissue and skeletal muscle, BAs induce energy expenditure by locally activating the type II iodothyronine deiodinase (DIO2), which transforms the inactive thyroxine (T4) to active thyroid hormone (T3), a key regulator of metabolism and energy homeostasis (125, 126). BAs seem to be the mediator of diet–induced thermogenesis by activating the BA–GPBAR–1– cAMP–DIO2 (type II iodothyronine deiodinase) signaling pathway. Diet–induced thermogenesis is probably impaired in obesity. BAs stimulation of brown adipocytes and myocytes increases their oxygen consumption, but this thermogenic effect is lost in DIO2 knockout mice (125).

An interesting model of bariatric surgery is represented by ileal interposition with or without Vertical sleeve gastrectomy (VSG). After this surgical procedure, obese Zucker rats display increased levels of circulating BAs. The intestinal adaptation after bariatric surgery is associated with increased recycling of BAs, which plays a protective role against obesity–related comorbidities (129, 136).

Similar effects are observed in rats with diet–induced obesity (130). Data obtained from the Ussing chamber (an electro–physiologic experimental model) show that BAs bind GPBAR–1 located in the basolateral membrane of the GLP–1–secreting L–cells. However, the GLP–1 release might occur only after the initial BA absorption across the intestinal epithelium (133) and effective stimulation of GPBAR–1. A translational study performed on rats and patients demonstrates that endothelial dysfunction which is typically found in obesity is rapidly reversed by gastric bypass according to Roux (RYGBP), which is able to induce a GLP–1–mediated restoration of the endothelium–protective properties of HDL (137).

VSG is a surgical procedure that is associated with an increase in serum BAs and a decrease in gene expression of the GPBAR–1 receptor in the white adipose tissue, independent from dietetic variations (138). The effects of RYGBP on BAs were extensively studied in morbidly obese subjects. Total fasting BAs display a bimodal rise after RYGB: ursodeoxycholic acid (UDCA) and its glycine and taurine conjugate increase after 1 month (with insulin–sensitizing effects), while primary unconjugated BAs, as well as deoxycholic acid and its glycine conjugate increases after 24 months (139–141). According to Watanabe et al., the increase in the circulating BA levels after bariatric surgery might activate the thyroid hormone–stimulated pathway involving TGR5 (or GPBAR–1) and enhance thermogenesis and weight loss (126).

When BAs bind ileal GPBAR–1, peptide YY (PYY) circulating levels as well as GLP–1 and GLP–2 increase playing an anorexigenic effect (i.e., appetite reduction), as well as GLP–1 and GLP–2 (142). An additional mechanism is the activation of the BA–GPBAR–1–GLP–1 axis which is followed by the GLP–1–induced insulin release, the decrease in glucagon secretion from the liver, and the inhibition of gastrointestinal motility and food assumption (143). In the same line of evidence, Yu, et al. demonstrated that the levels of chenodeoxycholic acid (CDCA) in obese patients with type 2 diabetes undergoing RYGBP might be considered a prognostic marker of diabetes remission after this bariatric procedure (144). To explain these results, the additional BAs signaling involving FXR might be taken into account. Shen, et al. reported that CDCA is able to increase intracellular glucose transport in adipocyte cell lines by activating GLUT4 transcription *via* the FXR–FXR response element (FXRE) signaling (145). The same mechanisms might control GLUT4 transcription in hepatocytes and ileal cells where BAs also act as signaling agents of the nuclear receptor FXR. After bariatric surgery, BAs/FXR axis might also clarify several effects on glucose and lipid metabolism (146). Düfer and co–workers showed that BAs/FXR interaction might acutely increase β–cell function and insulin secretion (147).

The gut microbiota might also be involved in this crosstalk between BAs and glucose and lipid metabolism (140). In obese FXR knockout mice, the beneficial effects of VSG are not completely explained by the mechanical restriction of the stomach per se but are also due to both increased circulating levels of BAs and associated changes in gut microbiota. Furthermore, the surgery–induced reduction of body weight and the improvement in glucose tolerance is blunted after the silencing of FXR (146).

Gut microbiota is responsible for the deconjugation, oxidation, sulfation, and dihydroxylation of the primary BAs to the secondary BAs (**Figure 1**). The possibility exists that the modified gut microbiota following bariatric surgery may modify the BAs’ composition and kinetic and metabolic function. Several data demonstrate that it is possible to transfer the gut microbiota from mice with RYGBP to germ–free mice, inducing body weight loss in germ–free mice. This result and others support the view that gut microbiota composition plays an important role in the beneficial effects observed after RYGBP. The overall mechanisms responsible for the increase in plasma BA concentrations after bariatric surgery is not completely clarified. Moreover, not all studies on bariatric surgery are in favor of the beneficial effect of BAs on glucose homeostasis and energy metabolism.

Kohli, et al. (148) found that RYGBP is indeed associated with increased circulating BAs and GPBAR–1 signaling (i.e., the increased peak of GLP–1 after a meal and decreased serum TSH). BAs do not appear to be simultaneous to the early increment in GLP–1 and gut peptide secretion in obese subjects undergoing bariatric surgery (RYGBP or VSG). GLP–1 and PYY circulating levels increase significantly 1 week and 3 months after the surgery. By contrast, both basal and postprandial levels of BAs increase more slowly and progressively, reaching significant rises only one year after the surgery (149). Unexpectedly, changes in circulating levels of BAs do not correlate with meal–mediated insulin response, insulin sensitivity, or basal thermogenesis.

Robust data show that the beneficial metabolic effects on glucose tolerance might occur by ileal interposition without intestinal resection (150–155). In this procedure, a part of the terminal ileum is interposed into the proximal jejunum. This puts nutrients prematurely in contact with the ileal mucosa and stimulates the L cells to produce GLP–1 and PYY. The increased delivery of BAs to the distal L cells and the altered GPBAR–1 receptor activation, however, do not seem to play a master role in the early increases in the intestinal secretion of GLP–1 and PYY, as seen after other procedures of bariatric surgery (156–163).

A list of the most studied hepatokines is reported in **Table 1**. Recent studies show that Fetuin–B plays additional roles through its interaction with Farnesoid X Receptor (FXR or NR1H4) (**Figure 1**). In studies utilizing microarray technology, Fetuin–B acts as an FXR agonist–regulated gene. The interaction between BAs and Fetuin–B needs further studies in humans, especially in models of ketogenic diets, because they activate the same nuclear receptor pathway. However, from the present data, it seems that the connecting point between hepatokines, BAs, and KBs is represented by the liver itself but in the contest of the microbiota–gut–brain system.

**Table 1:** List of some of the hepatokines expressed in humans.

Name	Target organs	Role	Circulating levels	Reference
ANGPTL3	Liver, Ads, SkM, Brain, Heart	increases InsR inhibits LPL inactives LPL	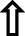	(164–169)
ANGPTL6	Several	regulates InsS regulates glucose and lipid metabolism	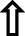	(42–44, 170, 171)
FIAF (ANGPTL4)	Liver, Ads, SkM, Brain	increases glucose production	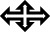	(49–51, 54, 172–174)
Fetuin A Fetuin B	Liver, Ads, SkM Liver, SkM,	inhibits insulin receptor phosphorylation increases InsR increases AdT inflammation inhibits insulin receptor phosphorylation interacts with FXR	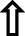 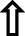	(28, 33, 63–65)
FGF21	Ads, Brain	increases EE increases InsS decreases sugar intake decreases circulating TGL	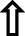	(72, 175–177)
Lipasin (ANGPTL8)	Liver, Ads, SkM, Brain, Heart	increases InsR inhibits LPL binds ANGPTL3 downregulates FIAF	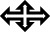	(3, 178, 179)
Selenoprotein P	Several	increases glucose utilization impairs Insulin signaling	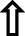	(180, 181)

Ads, adipocytes; ANGPTL, Angiopoietin–like proteins; BW, Body weight; EE, Energy expenditure; InsR, Insulin resistance; InsS, Insulin sensitivity; SkM, Skeletal muscles. 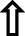 Increased 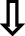 decreased 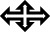 contrasting results.

## Conclusions

The liver has recently acquired the dignity of an endocrine organ, especially for its ability to express hepatokines. Some hepatokines are promising markers of metabolic abnormalities. The liver is also involved in KBs and BAs’ production. Both KBs and BAs are involved in endocrine, paracrine, and autocrine effects already confirmed in humans. Analogs for some hepatokine receptors are gradually being valued and could become new therapeutic approaches for metabolic diseases. BAs might probably be considered with major attention in the context of obesity prevention, treatment, and management because they play an important role in modulating the microbiota–brain interaction as suggested by studies performed in animal models of bariatric surgery and humans undergoing bariatric metabolic surgery. The current treatment of type 2 diabetes with inhibitors of tubular sodium/glucose co–transporter–2 (GLT2i) highlighted additional important therapeutic roles played by KBs.

## Author contributions

GG wrote the review and prepared the figure, JB revised the manuscript, AC added references, SP revised the manuscript, FG critically revised the manuscript. All authors contributed to the article and approved the submitted version.

## References

[1] Ravelli AC van der Meulen JH Osmond C Barker DJ Bleker OP. Obesity at the age of 50 y in men and women exposed to famine prenatally. Am J Clin Nutr (1999) 70:811– 6. doi: 10.1093/ajcn/70.5.811 10539740

[2] Di Ciaula A Portincasa P. Fat, epigenome and pancreatic diseases. interplay and common pathways from a toxic and obesogenic environment. Eur J Intern Med (2014) 25:865–73. doi: 10.1016/j.ejim.2014.10.012 25457435

[3] Bolton JL Smith SH Huff NC Gilmour MI Foster WM Auten RL. Prenatal air pollution exposure induces neuroinflammation and predisposes offspring to weight gain in adulthood in a sex–specific manner. FASEB J (2012) 26:4743–54. doi: 10.1096/fj.12-210989 22815382

[4] Lind PM Roos V Ronn M Johansson L Ahlstrom H Kullberg J. Serum concentrations of phthalate metabolites are related to abdominal fat distribution two years later in elderly women. Environ Health (2012) 11:21. doi: 10.1186/1476-069X-11-21 22472124 PMC3379932

[5] Fenichel P Chevalier N Brucker–Davis F. Bisphenol a: an endocrine and metabolic disruptor. Ann Endocrinol (Paris) (2013) 74:211–20. doi: 10.1016/j.ando.2013.04.002 23796010

[6] Li DK Miao M Zhou Z Wu C Shi H Liu X. Urine bisphenol–a level in relation to obesity and overweight in school–age children. PloS One (2013) 8:e65399. doi: 10.1371/journal.pone.0065399 23776476 PMC3680397

[7] Smink A Ribas–Fito N Garcia R Torrent M Mendez MA Grimalt JO. Exposure to hexachlorobenzene during pregnancy increases the risk of overweight in children aged 6 years. Acta Paediatr (2008) 97:1465–9. doi: 10.1111/j.1651-2227.2008.00937.x 18665907

[8] Rundle A Hoepner L Hassoun A Oberfield S Freyer G Holmes D. Association of childhood obesity with maternal exposure to ambient air polycyclic aromatic hydrocarbons during pregnancy. Am J Epidemiol (2012) 175:1163– 72. doi: 10.1093/aje/kwr455 PMC3491973 22505764

[9] Ortiz L Nakamura B Li X Blumberg B Luderer U. *In utero* exposure to benzo[a]pyrene increases adiposity and causes hepatic steatosis in female mice, and glutathione deficiency is protective. Toxicol Lett (2013) 223:260–7. doi: 10.1016/j.toxlet.2013.09.017 PMC3856224 24107266

[10] Thiering E Bruske I Kratzsch J Thiery J Sausenthaler S Meisinger C. Prenatal and postnatal tobacco smoke exposure and development of insulin resistance in 10–year old children. int J induce energy expenditure by promoting intracellular thyroid hormone activation. Nature (2006) 439:484–9. doi: 10.1038/nature04330

[11] Jerrett M McConnell R Wolch J Chang R Lam C Dunton G. Traffic–related air pollution and obesity formation in children: a longitudinal, multilevel analysis. Environ Health (2014) 13:49. doi: 10.1186/1476-069X-13-49 24913018 PMC4106205

[12] Kelishadi R Mirghaffari N Poursafa P Gidding SS. Lifestyle and environmental factors associated with inflammation, oxidative stress and insulin resistance in children. Atherosclerosis (2009) 203:311–9. doi: 10.1016/j.atherosclerosis.2008.06.022 18692848

[13] Thiering E Cyrys J Kratzsch J Meisinger C Hoffmann B Berdel D. Long–term exposure to traffic–related air pollution and insulin resistance in children: results from the GINIplus and LISA plus birth cohorts. Diabetologia (2013) 56:1696–704. doi: 10.1007/s00125-013-2925-x PMC3699704 23666166

[14] Stanner SA Yudkin JS. Fetal programming and the Leningrad siege study. Twin Res (2001) 4:287–92. doi: 10.1375/twin.4.5.287 11913363

[15] Shi H Su B. Molecular adaptation of modern human populations. Int J Evol Biol 2010 Dec 30 (2011) 484769. doi: 10.4061/2011/484769 PMC3039432 21350631

[16] Heijmans BT Tobi EW Stein AD Putter H Blauw GJ Susser ES. Persistent epigenetic differences associated with prenatal exposure to famine in humans. Proc Natl Acad Sci USA (2008) 105:17046–9. doi: 10.1073/pnas.0806560105 PMC2579375 18955703

[17] Swinburn BA Sacks G Hall KD McPherson K Finegood DT Moodie ML. The global obesity pandemic: shaped by global drivers and local environments. Lancet (2011) 378: Hyg Environ Health (2011) 214:361–8. doi: 10.1016/j.ijheh.2011.04.004 21872749

[18] Egger G Dixon J. Non–nutrient causes of low–grade, systemic inflammation: support for a ‘canary in the mineshaft’ view of obesity in chronic disease. Obes Rev (2011) 12:339–45. doi: 10.1111/j.1467-789X.2010.00795.x 20701689

[19] Kelishadi R Mirghaffari N Poursafa P Gidding SS. Lifestyle and environmental factors associated with inflammation, oxidative stress and insulin resistance in children. Atherosclerosis (2009) 203:311–9. 10.1016/j.atherosclerosis.2008.06.022 18692848

[20] Egger G Dixon J. Non-nutrient causes of low-grade, systemic inflammation: support for a ‘canary in the mineshaft’ view of obesity in chronic disease. Obes Rev (2011) 12:339–45. doi: 10.1111/j.1467-789X.2010.00795.x 20701689

[21] Ruiz-Nunez B Pruimboom L Dijck-Brouwer DA Muskiet FA. Lifestyle and nutritional imbalances associated with western diseases: causes and consequences of chronic systemic low-grade inflammation in an evolutionary context. J Nutr Biochem (2013) 24:1183–201. doi: 10.1016/j.jnutbio.2013.02.009 23657158

[22] Hotamisligil,GS. Inflammation and metabolic disorders. Nature (2006) 444(7121):860e867. doi: 10.1038/nature05485 17167474

[23] Roden M Bernroider E. Hepatic glucose metabolism in humans and its role in health and disease. Best Pract Res Clin Endocrinol Metab (2003) 17(3):365–83. doi: 10.1016/S1521-690X(03)00031-9 12962691

[24] Leone TC Weinheimer CJ Kelly DP. A critical role for the peroxisome proliferatoractivated receptor alpha (PPARalpha) in the cellular fasting response: the PPARalpha-null mouse as a model of fatty acid oxidation disorders. Proc Natl Acad Sci USA (1999) 96(13):7473–8. doi: 10.1073/pnas.96.13.7473 PMC22110 10377439

[25] Di Ciaula A Garruti G Lunardi Baccetto R Molina-Molina E Bonfrate L Wang DQH. Piero portincasa. Bile Acid Physiol Ann Hepatol (2017) 16(1):s4–s14. 10.5604/01.3001.0010.5493 29080336

[26] Jensen-Cody SO Potthoff MJ. Hepatokines and metabolism: Deciphering communication from the liver. Mol Metab (2021) 44:101138. doi: 10.1016/j.molmet.2020.10113 33285302 PMC7788242

[27] Garruti G Wang HH Bonfrate L de Bari O Wang DQH Portincasa PA. Pleiotropic role for the orphan nuclear receptor small heterodimer partner in lipid homeostasis and metabolic pathways. J Lipids (2012) 2012:2. doi: 10.1155/2012/304292 PMC3346990 22577560

[28] Stefan N Fritsche A Weikert C Boeing H Joost HG Haring HU. Plasma fetuin-a levels and the risk of type 2 diabetes. Diabetes (2008) 57(10):2762–7. doi: 10.2337/db08-0538 PMC2551687 18633113

[29] Pocai A Obici S Schwartz GJ Rossetti L. A brain-liver circuit regulates glucose homeostasis. Cell Metab (2005) 1(1):53–61. doi: 10.1016/j.cmet.2004.11.001 16054044

[30] Cahill GF Jr. Fuel metabolism in starvation. Annu Rev Nutr (2006) 26:1–22. doi: 10.1146/annurev.nutr.26.061505.111258 16848698

[31] McGarry JD Foster DW. Regulation of hepatic fatty acid oxidation and ketone body production. Annu Rev Biochem (1980) 49:395–420. doi: 10.1146/annurev.bi.49.070180.002143 6157353

[32] Meex RC Watt MJ. Hepatokines: linking non-alcoholic fatty liver disease and insulin resistance. Nat Rev Endocrinol (2017) 13:509–20. doi: 10.1038/nrendo.2017.56 28621339

[33] Meex RC Hoy AJ Morris A Brown RD Lo JCY Burke M. Fetuin b is a secreted hepatocyte factor linking steatosis to impaired glucose metabolism. Cell Metab (2015) 22:1078–89. doi: 10.1016/j.cmet.2015.09.023 26603189

[34] Hato T Tabata M Oike Y. The role of angiopoietin-like proteins in angiogenesis and metabolism. Trends Cardiovasc Med (2008) 18(1):6–14. doi: 10.1016/j.tcm.2007.10.003 18206803

[35] Romeo S Yin W Kozlitina J Pennacchio LA Boerwinkle E Hobbs HH. Rare loss-offunction mutations in ANGPTL family members contribute to plasma triglyceride levels in humans. J Clin Invest (2009) 119(1):70–9. 10.1172/JCI37118 PMC2613476 19075393

[36] Minicocci I Montali A Robciuc MR Quagliarini F Censi V Labbadia G. Mutations in the ANGPTL3 gene and familial combined hypolipidemia: a clinical and biochemical characterization. J Clin Endocrinol Metab (2012) 97(7):E1266–75. doi: 10.1210/jc.2012-1298 PMC5393441 22659251

[37] Shimizugawa T Ono M Shimamura M Yoshida K Ando Y Koishi R. ANGPTL3 decreases very low-density lipoprotein triglyceride clearance by inhibition of lipoprotein lipase. J Biol Chem (2002) 277(37):33742–33748. doi: 10.1074/jbc.M203215200 12097324

[38] Musunuru K Pirruccello JP Do R Peloso GM Guiducci C. Exome sequencing, ANGPTL3 mutations, and familial combined hypolipidemia. New Engl J Med (2010) 363(23):2220–7. doi: 10.1056/NEJMoa1002926 PMC3008575 20942659

[39] Wang Y McNutt MC Banfi S Levin MG Holland WL Gusarova V. Hepatic ANGPTL3 regulates adipose tissue energy homeostasis. Proc Natl Acad Sci U.S.A. (2015) 112(37):11630–5. doi: 10.1073/pnas.1515374112 PMC4577179 26305978

[40] Duntas LH Brenta G. A renewed focus on the association between thyroid hormones and lipid metabolism. Front Endocrinol (2018) 9:511. doi: 10.3389/fendo.2018.00511 PMC6129606 30233497

[41] Pramfalk C Parini P Gustafsson U Sahlin S Eriksson M. Effects of high-dose statin on the human hepatic expression of genes involved in carbohydrate and triglyceride metabolism. J Internal Med (2011) 269(3):333–9. doi: 10.1111/j.1365-2796.2010.02305.x 21083855

[42] Raal FJ Rosenson RS Reeskamp LF Hovingh GK Kastelein JJP Rubba P. Evinacumab for homozygous familial hypercholesterolemia. New Engl J Med (2020) 383(8):711–20. doi: 10.1056/NEJMoa2004215 32813947

[43] Yilmaz Y Ulukaya E Atug O Dolar E. Serum concentrations of human angiopoietin-like protein 3 in patients with nonalcoholic fatty liver disease: association with insulin resistance. Eur J Gastroenterol Hepatol (2009) 21(11):1247–51. doi: 10.1097/MEG.0b013e32832b77ae 19474742

[44] Oike Y Akao M Yasunaga K Yamauchi T Morisada T Ito Y. Angiopoietin-related growth factor antagonizes obesity and insulin resistance. Nat Med (2005) 11(4):400–408. doi: 10.1038/nm1214 15778720

[45] Kitazawa M Ohizumi Y Oike Y Hishinuma T Hashimoto S. Angiopoietin-related growth factor suppresses gluconeogenesis through the akt/forkhead box class O1-dependent pathway in hepatocytes. J Pharmacol Exp Ther (2007) 323(3):787e793. doi: 10.1124/jpet.107.127530 17804676

[46] Qaddoumi MG Alanbaei M Hammad MM Al Khairi I Cherian P Channanath A. Investigating the role of myeloperoxidase and angiopoietin-like protein 6 in obesity and diabetes. Sci Rep (2020) 10(1):6170. doi: 10.1038/s41598-020-63149-7 32277104 PMC7148302

[47] Ebert T Bachmann A Lossner U Kratzsch J Bluher M Stumvoll M. Serum levels of angiopoietin-related growth factor in diabetes mellitus and chronic hemodialysis. Metabolism (2009) 58(4):547–551. doi: 10.1016/j.metabol.2008.11.016 19303977

[48] Namkung J Koh SB Kong ID Choi JW Yeh BI. Serum levels of angiopoietin-related growth factor are increased in metabolic syndrome. Metabolism (2011) 60(4):564e568. doi: 10.1016/j.metabol.2010.05.013 20673930

[49] Mattijssen F Alex S Swarts HJ Groen AK van Schothorst EM Kersten S. Angptl4 serves as an endogenous inhibitor of intestinal lipid digestion. Mol Metab (2014) 3(2):135–144. doi: 10.1016/j.molmet.2013.11.004 24634819 PMC3953698

[50] Dijk W Heine M Vergnes L Boon MR Schaart G Hesselink MK. ANGPTL4 mediates shuttling of lipid fuel to brown adipose tissue during sustained cold exposure. eLife (2015 2015) 4:e08428. doi: 10.7554/eLife.08428 26476336 PMC4709329

[51] Cushing EM Chi X Sylvers KL Shetty SK Potthoff MJ Davies BSJ. Angiopoietin-like 4 directs uptake of dietary fat away from adipose during fasting. Mol Metab (2017) 6(8):809–18. doi: 10.1016/j.molmet.2017.06.007 PMC5518724 28752045

[52] Yoshida K Shimizugawa T Ono M Furukawa H. Angiopoietin-like protein 4 is a potent hyperlipidemia-inducing factor in mice and inhibitor of lipoprotein lipase. J Lipid Res (2002) 43(11):1770–2. doi: 10.1194/jlr.C200010-JLR200 12401877

[53] Cinkajzlova A Mraz M Lacinova Z Klouckova J Kavalkova P Kratochvilova H. Angiopoietin-like protein 3 and 4 in obesity, type 2 diabetes mellitus, and malnutrition: the effect of weight reduction and realimentation. Nutr Diabetes (2018) 8(1):21. doi: 10.1038/s41387-018-0032-2 29695708 PMC5916880

[54] Sukonina V Lookene A Olivecrona T Olivecrona G. Ngiopoietinlike protein 4 converts lipoprotein lipase to inactive monomers and modulates lipase activity in adipose tissue. Proc Natl Acad Sci U.S.A. (2006) 103(46):17450–5. doi: 10.1073/pnas.0604026103 PMC1859949 17088546

[55] Gonzalez-Gil AM Elizondo-Montemayor L. The role of exercise in the interplay between myokines, hepatokines, osteokines, adipokines, and modulation of inflammation for energy substrate redistribution and fat mass loss: a review. Nutrients (2020) 12(6):1899. doi: 10.3390/nu12061899 32604889 PMC7353393

[56] Xu A Lam MC Chan KW Wang Y Zhang J Hoo RL. Angiopoietin-like protein 4 decreases blood glucose and improves glucose tolerance but induces hyperlipidemia and hepatic steatosis in mice. Proc Natl Acad Sci U.S.A. (2005) 102(17):6086–91. doi: 10.1073/pnas.0408452102 PMC1087912 15837923

[57] Tjeerdema N Georgiadi A Jonker JT van Glabbeek M Alizadeh Dehnavi R Tamsma JT. Inflammation increases plasma angiopoietin-like protein 4 in patients with the metabolic syndrome and type 2 diabetes. BMJ Open Diabetes Res Care (2014) 2(1):e000034. doi: 10.1136/bmjdrc-2014-000034 PMC4265148 25512873

[58] Desai U Lee EC Chung K Gao C Gay J Key B. Lipid lowering effects of anti-angiopoietin-like-4 antibody recapitulate the lipid phenotype found in angiopoietin-like 4 knockout mice. Proc Natl Acad Sci U.S.A. (2007) 104(28):11766–71. doi: 10.1073/pnas.0705041104 PMC1913890 17609370

[59] Watt MJ Miotto PM De Nardo W Montgomery MK. The liver as an endocrine organlinking NAFLD and insulin resistance. Endocrine Rev (2019 2019) 40(5):1367–93. doi: 10.1210/er.2019-00034 31098621

[60] Schafer C Heiss A Schwarz A Westenfeld R Ketteler M Floege J. The serum protein α2-Heremans–schmid glycoprotein/fetuin-a is a systemically acting inhibitor of ectopic calcification. J Clin Invest (2003) 112:357–66. doi: 10.1172/JCI17202 PMC166290 12897203

[61] Schinke T Amendt C Trindl A Poschke O. Muller-esterl w and jahnen-dechent w. the serum protein α2-HS glycoprotein/fetuin inhibits apatite formation in vitro and in mineralizing calvaria cells. a possible role in mineralization and calcium homeostasis. J Biol Chem (1996) 271:20789–96. doi: 10.1074/jbc.271.34.20789 8702833

[62] Szweras M Liu D Partridge EA Pawling J Sukhu B Clokie C. α2-HS glycoprotein/fetuin, a transforming growth factor-β/bone morphogenetic protein antagonist, regulates postnatal bone growth and remodeling. J Biol Chem (2002) 277:19991–7. doi: 10.1074/jbc.M112234200 11901155

[63] Mathews ST Singh GP Ranalletta M Cintron VJ Qiang X Goustin AS. Improved insulin sensitivity and resistance to weight gain in mice null for the ahsg gene. Diabetes (2002) 51(8):2450–8. doi: 10.2337/diabetes.51.8.2450 12145157

[64] Goustin AS Abou-Samra AB. The "thrifty" gene encoding Ahsg/Fetuin-a meets the insulin receptor: insights into the mechanism of insulin resistance. Cell Signalling (2011) 23(6):980 –990. doi: 10.1016/j.cellsig.2010.11.003 21087662

[65] Stefan N Haring HU. The role of hepatokines in metabolism. Nat Rev Endocrinol (2013) 9(3):144–52. doi: 10.1038/nrendo.2012.258 23337953

[66] Karmilin K Schmitz C Kuske M Körschgen H Olf M Meyer K. Sci Rep (2019) 9:546. doi: 10.1038/s41598-018-37024-5 30679641 PMC6346019

[67] Forman BM Goode E Chen J Oro AE Bradley DJ Perlmann T. Identification of a nuclear receptor that is activated by farnesol metabolites. Cell (1995) 81:687–93. doi: 10.1016/0092-8674(95)90530-8 7774010

[68] Seol W Choi HS. And moore DD isolation of proteins that interact specifically with the retinoid x receptor: two novel orphan receptors. Mol Endocrinol (1995) 9:72–85. 7760852 10.1210/mend.9.1.7760852

[69] Garruti G Di Ciaula A Wang HH Wang DQH Portincasa P. Cross-talk between bile acids and thermogenic and gastro-intestinal hormones. Ann Hepatol (2017) 16(Suppl. 1):s68–82. doi: 10.5604/01.3001.0010.5499 29080342

[70] Pan X Wen SW Bestman PL Kaminga AC Acheampong K Liu A. Fetuin-a in metabolic syndrome: A systematic review and meta-analysis. PloS One (2020) 15(3):e0229776. doi: 10.1371/journal.pone.0229776 32134969 PMC7058339

[71] Choi KM Han KA Ahn HJ Lee SY Hwang SY Kim BH. The effects of caloric restriction on fetuin-a and cardiovascular risk factors in rats and humans: a randomized controlled trial. Clin Endocrinol (2013) 79(3):356e363. doi: 10.1111/cen.12076 23067229

[72] Ochi A Mori K Emoto M Nakatani S Morioka T Motoyama K. Direct inhibitory effects of pioglitazone on hepatic fetuin-a expression. PloS One (2014) 9(2):e88704. doi: 10.1371/journal.pone.0088704 24551137 PMC3923806

[73] Badman MK Pissios P Kennedy AR Koukos G Flier JS Maratos-Flier E. Hepatic fibroblast growth factor 21 is regulated by PPARalpha and is a key mediator of hepatic lipid metabolism in ketotic states. Cell Metab (2007) 5(6):426e437. doi: 10.1016/j.cmet.2007.05.002 17550778

[74] Andersen B Straarup EM Heppner KM Takahashi DL Raffaele V Dissen GA. FGF21 decreases body weight without reducing food intake or bone mineral density in high-fat fed obese rhesus macaque monkeys. Int J Obes (2018) 42(6):1151e1160. doi: 10.1038/s41366-018-0080-7 PMC6733401 29892039

[75] Coskun T Bina HA Schneider MA Dunbar JD Hu CC Chen Y. Fibroblast growth factor 21 corrects obesity in mice. Endocrinology (2008 2008) 149(12):6018–6027. doi: 10.1210/en.2008-0816 18687777

[76] Xu J Lloyd DJ Hale C Stanislaus S Chen M Sivits G. Fibroblast growth factor21 reverses hepatic steatosis, increases energy expenditure, and improves insulin sensitivity in diet-induced obese mice. Diabetes (2009) 58(1):250–259. doi: 10.2337/db08-0392 18840786 PMC2606881

[77] Kharitonenkov A Shiyanova TL Koester A Ford AM Micanovic R Galbreath EJ. FGF21 as a novel metabolic regulator. J Clin Invest (2005) 115(6):1627e1635. 15902306 10.1172/JCI23606 PMC1088017

[78] Yan J Nie Y Cao J Luo M Yan M Chen Z and He B. The roles and pharmacological effects of FGF21 in preventing aging-associated metabolic diseases. Front Cardiovasc Med (2021) 8:65557. doi: 10.3389/fcvm.2021.65557 PMC8044345 33869312

[79] Hill CM Laeger T Dehner M Albarado DC Clarke B Wanders D. FGF21 signals protein status to the brain and adaptively regulates food choice and metabolism. Cell Rep (2019) 27(10):2934–2947. doi: 10.1016/j.celrep.2019.05.022 31167139 PMC6579533

[80] Schumann G Liu C O’Reilly P Gao H Song P Xu B. KLB is associated with alcohol drinking, and its gene product beta-klotho is necessary for FGF21 regulation of alcohol preference. Proc Natl Acad Sci U.S.A. (2016) 113(50):14372e14377. 27911795 10.1073/pnas.1611243113 PMC5167198

[81] Gaich G Chien JY Fu H Glass LC Deeg MA Holland WL. The effects of LY2405319, an FGF21 analog, in obese human subjects with type 2 diabetes. Cell Metab (2013) 18(3):333–340. doi: 10.1016/j.cmet.2013.08.005 24011069

[82] Sa-Nguanmoo P Chattipakorn N Chattipakorn SC. Potential roles of fibroblast growth factor 21 in the brain. Metab Brain Dis (2016) 31(2):239–48. doi: 10.1007/s11011-015-9789-3 26738728

[83] Quagliarini F Wang Y Kozlitina J Grishin NV Hyde R Boerwinkle E. Atypical angiopoietin-like protein that regulates ANGPTL3. Proc Natl Acad Sci U.S.A. (2012) 109(48):19751e19756. doi: 10.1073/pnas.1217552109 23150577 PMC3511699

[84] Wang R Yuan J Zhang C Wang L Liu Y Song L. Neuropeptide y-positive neurons in the dorsomedial hypothalamus are involved in the anorexic effect of Angptl8. Front Mol Neurosci (2018) 11:451–55. doi: 10.3389/fnmol.2018.00451 PMC6305345 30618603

[85] Hariharan S Dharmaraj S. Selenium and selenoproteins: it’s role in regulation of inflammation. Inflammopharmacology (2020) 28(3):667e695. doi: 10.1007/s10787-020-00690-x 32144521 PMC7222958

[86] Misu H Takamura T Takayama H Hayashi H Matsuzawa-Nagata N Kurita S. A liverderived secretory protein, selenoprotein p, causes insulin resistance. Cell Metab (2010) 12(5):483e495. 21035759 10.1016/j.cmet.2010.09.015

[87] Misu H Ishikura K Kurita S Takeshita Y Ota T Saito Y. Inverse correlation between serum levels of selenoprotein p and adiponectin in patients with type 2 diabetes. PloS One (2012) 7(4):e34952. doi: 10.1371/journal.pone.0034952 22496878 PMC3319626

[88] Yang SJ Hwang SY Choi HY Yoo HJ Seo JA Kim SG. Serum selenoprotein p levels in patients with type 2 diabetes and prediabetes: implications for insulin resistance, inflammation, and atherosclerosis. J Clin Endocrinol Metab (2011) 96(8):E1325–E1329. doi: 10.1210/jc.2011-0620 21677040

[89] Cox PJ Kirk T Ashmore T Willerton K Evans R Smith A. Nutritional ketosis alters fuel preference and thereby endurance performance in athletes. Cell Metab (2016) 24:256–68. doi: 10.1016/j.cmet.2016.07.010 27475046

[90] Puchalska P Crawford PA. Multi-dimensional roles of ketone bodies in fuel metabolism, signaling, and therapeutics. Cell Metab (2017) 25(2):262–84. doi: 10.1016/j.cmet.2016.12.022 PMC5313038 28178565

[91] Phinney SD Bistrian BR Wolfe RR Blackburn GL. The human metabolic response to chronic ketosis without caloric restriction: physical and biochemical adaptation. Metabolism (1983) 32(8):757–68. doi: 10.1016/0026-0495(83)90105-1 6865775

[92] Yancy WS Jr Olsen MK Guyton JR Bakst RP Westman EC. A low carbohydrate, ketogenic diet versus a low-fat diet to treat obesity and hyperlipidemia: a randomized, controlled trial. Ann Intern Med (2004) 140(10):769–77. doi: 10.7326/0003-4819-140-10-200405180-00006 15148063

[93] Watanabe M Tuccinardi D Ernesti I Basciani S Mariani S Genco A. Scientific evidence underlying contraindications to the ketogenic diet: An update. Obes Rev (2020) 21:e13053. doi: 10.1111/obr.13053 32648647 PMC7539910

[94] Bergqvist AG Chee CM Lutchka L Rychik J Stallings VA. Selenium deficiency associated with cardiomyopathy: a complication of the ketogenic diet. Epilepsia (2003) 44:618–20. doi: 10.1046/j.1528-1157.2003.26102.x 12681013

[95] Isner JM Sours HE Paris AL Ferrans VJ Roberts WC. Sudden, unexpected death in avid dieters using the liquid-protein-modified fast diet. observations in 17 patients and the role of the prolonged QT interval. Circulation (1979) 60(6):1401–12. 10.1161/01.cir.60.6.1401 498466

[96] Terzikhan N Doets EL Vonk N-SM. Extensive literature search and review as preparatory work for the evaluation of the essential composition of total diet replacement products for weight control. EFSA Supporting Publications (2015), EN–590. doi: 10.2903/j.efsa.2015.3957

[97] Zhang S Zhuang X Lin X. Low-carbohydrate diets and risk of incident atrial fibrillation: a prospective cohort study. J Am Heart Assoc (2019) 8(9):e011955. doi: 10.1161/JAHA.119.011955 31020911 PMC6512089

[98] Simeone TA Matthews SA Samson KK Simeone KA. Regulation of brain PPARgamma2 contributes to ketogenic diet anti-seizure efficacy. Exp Neurol (2017) 287:54–64. doi: 10.1016/j.expneurol.2016.08.006 27527983 PMC5110374

[99] Kose E Guzel O Demir K and Arsla N. Changes of thyroid hormonal status in patients receiving ketogenic diet due to intractable epilepsy. J Pediatr Endocrinol Metab (2017) 30(4):411–6. doi: 10.1515/jpem-2016-0281 28076316

[100] Bank IM Shemie SD Rosenblatt B Bernard C Mackie AS. Sudden cardiac death in association with the ketogenic diet. Pediatr Neurol (2008) 39(6):429–31. doi: 10.1016/j.pediatrneurol.2008.08.013 19027591

[101] Sirikonda NS Patten WD Phillips JR Mullett CJ. Ketogenic diet: rapid onset of selenium deficiency-induced cardiac decompensation. Pediatr Cardiol (2012) 33(5):834–8. doi: 10.1007/s00246-012-0219-6 22367552

[102] Castellana M Conte E Cignarelli A Perrini S Giustina A Giovanella L. Efficacy and safety of very low-calorie ketogenic diet (VLCKD) in patients with overweight and obesity: a systematic review and meta-analysis. Rev Endocr Metab Disord (2020) 21(1):5–16. doi: 10.1007/s11154-019-09514-y 31705259

[103] Aubert G Martin OJ Horton JL Lai L Vega RB Leone TC. The failing heart relies on ketone bodies as a fuel. Circulation (2016) 133:698–705. doi: 10.1161/CIRCULATIONAHA.115.017355 26819376 PMC4766035

[104] Longo N Fukao T Singh R Pasquali M Barrios RG Kondo N. Succinyl-CoA: 3-ketoacid transferase (SCOT) deficiency in a new patient homozygous for an R217X mutation. J Inherit Metab Dis (2004) 27:691–2. doi: 10.1023/B:BOLI.0000043023.57321.18 15669687

[105] Janardhan A Chen J Crawford PA. Altered systemic ketone body metabolism in advanced heart failure. Tex Heart Inst J (2011) 38:533–8. PMC3231554 22163128

[106] Bedi KC Snyder NW Brandimarto J Aziz M Mesaros C Worth AJ. Evidence for intramyocardial disruption of lipid metabolism and increased myocardial ketone utilization in advanced human heart failure. Circulation (2016) 133:706–16. doi: 10.1161/CIRCULATIONAHA.115.017545 PMC4779339 26819374

[107] Wang P Tate JM Lloyd SG. Low carbohydrate diet decreases myocardial insulin signaling and increases susceptibility to myocardial ischemia. Life Sci (2008) 83:836–44. doi: 10.1016/j.lfs.2008.09.024 PMC2642968 18951908

[108] Kupari M Lommi J Ventila M Karjalainen U. Breath acetone in congestive heart failure. Am J Cardiol (1995) 76:1076–8. doi: 10.1016/S0002-9149(99)80304-X 7484868

[109] Lommi J Kupari M Koskinen P Naveri H Leinonen H Pulkki K. Blood ketone bodies in congestive heart failure. J Am Coll Cardiol (1996) 28:665–72. doi: 10.1016/0735-1097(96)00214-8 8772754

[110] Suzuki M Takeda M Kito A Fukazawa M Yata T Yamamoto M. Tofogliflozin, a sodium/glucose cotransporter 2 inhibitor, attenuates body weight gain and fat accumulation in diabetic and obese animal models. Nutr Diabetes (2014) 4:e125. doi: 10.1038/nutd.2014.20 25000147 PMC5189930

[111] Inagaki N Goda M Yokota S Maruyama N Iijima H. Safety and efficacy of canagliflozin in japanese patients with type 2 diabetes mellitus: *post hoc* subgroup analyses according to body mass index in a 52-week open-label study. Expert Opin Pharmacother (2015) 16:1577–91. doi: 10.1517/14656566.2015.1055250 26104600

[112] Ferrannini E Baldi S Frascerra S Astiarraga B Heise T Bizzotto R. Shift to fatty substrate utilization in response to sodium-glucose cotransporter 2 inhibition in subjects without diabetes and patients with type 2 diabetes. Diabetes (2016) 65:1190–5. doi: 10.2337/db15-1356 26861783

[113] Zinman B Wanner C Lachin JM Fitchett D Bluhmki E Hantel S. Empagliflozin, cardiovascular outcomes, and mortality in type 2 diabetes. N Engl J Med (2015) 373:2117–28. doi: 10.1056/NEJMoa1504720 26378978

[114] Fitchett D Zinman B Wanner C Lachin JM Hantel S Salsali A. EMPA-REG OUTCOME(R) trial investigators. heart failure outcomes with empagliflozin in patients with type 2 diabetes at high cardiovascular risk: results of the EMPAREG OUTCOME(R) trial. Eur Heart J (Oxford) (2016) 37:1526–34. doi: 10.1093/eurheartj/ehv728 PMC4872285 26819227

[115] Vallon V Thomson SC. Targeting renal glucose reabsorption to treat hyperglycaemia: the pleiotropic effects of SGLT2 inhibition. Diabetologia (2017) 60(2):215–25. doi: 10.1007/s00125-016-4157-3 PMC5884445 27878313

[116] Li T Chiang JYL. Bile acid signaling in metabolic disease and drug therapy. Pharmacol Rev (2014) 66:948–83. doi: 10.1124/pr.113.008201 PMC4180336 25073467

[117] Wang DQH Neuschwander-Tetri BA Portincasa P. The biliary system. 2nd Ed. Morgan & Claypool Life Sciences (2017).

[118] Schalm SW LaRusso NF Hofmann AF Hoffman NE van Berge-Henegouwen GP Korman MG. Diurnal serum levels of primary conjugated bile acids. assessment by specific radioimmunoassays for conjugates of cholic and chenodeoxycholic acid. Gut (1978) 19:1006– 14. doi: 10.1136/gut.19.11.1006 PMC1412237 569619

[119] LaRusso NF Korman MG Hoffman NE Hofmann AF. Dynamics of the enterohepatic postprandial serum concentrations of conjugates of cholic acid in health, cholecystectomized patients, and patients with bile acid malabsorption. N Engl J Med (1974) 291:689–92. doi: 10.1056/NEJM197410032911401 4851463

[120] Ridlon JM Kang DJ Hylemon PB. Bile salt biotransformations by human intestinal bacteria. J Lipid Res (2006) 47:241–59. doi: 10.1194/jlr.R500013-JLR200 16299351

[121] Zhou H Hylemon PB. Bile acids are nutrient signaling hormones. Steroids (2014) 86:62–8. doi: 10.1016/j.steroids.2014.04.016 PMC4073476 24819989

[122] Forman BM Goode E Chen J Oro AE Bradley DJ Perlmann T. Identification of a nuclear receptor that is activated by farnesol metabolites. Cell (1995) 81:687–93. doi: 10.1016/0092-8674(95)90530-8 7774010

[123] Seol W Choi HS. And moore DD isolation of proteins that interact specifically with the retinoid x receptor: two novel orphan receptors. Mol Endocrinol (1995) 9:72–85. 7760852 10.1210/mend.9.1.7760852

[124] Ma K Saha PK Chan L and Moore DD. Farnesoid x receptor is essential for normal glucose homeostasis. J Clin Invest (2006) 116:1102–9. doi: 10.1172/JCI25604 PMC1409738 16557297

[125] Ma K Saha PK Chan L and Moore DD. Farnesoid x receptor is essential for normal glucose homeostasis. J Clin Invest (2006) 116:1102–9. doi: 10.1172/JCI25604 PMC1409738 16557297

[126] Cariou B van Harmelen K Duran-Sandoval D van Dijk TH Grefhorst A Abdelkarim M. The farnesoid x receptor modulates adiposity and peripheral insulin sensitivity in mice. J Biol Chem (2006 2006) 281:11039–49. doi: 10.1074/jbc.M510258200 16446356

[127] Yamagata K Daitoku H Shimamoto Y Matsuzaki H Hirota K Ishida J. Bile acids regulate gluconeogenic gene expression *via* small heterodimer partner-mediated repression of hepatocyte nuclear factor 4 and Foxo1. J Biol Chem (2004) 279:23158–65. doi: 10.1074/jbc.M314322200 15047713

[128] Duran-Sandoval D Cariou B Percevault F Hennuyer N Grefhorst A van Dijk TH. The farnesoid x receptor modulates hepatic carbohydrate metabolism during the fasting–refeeding transition. J Biol Chem (2005) 280:29971–9. doi: 10.1074/jbc.M501931200 15899888

[129] Makishima M Okamoto AY Repa JJ Tu H Learned RM Luk A. Identification of a nuclear receptor for bile acids. Science (1999) 284:1362–5. doi: 10.1126/science.284.5418.1362 10334992

[130] Kuipers F Bloks VW Groen AK. Beyond intestinal soap— bile acids in metabolic control. Nat Rev Endocrinol (2014) 10:488–98. doi: 10.1038/nrendo.2014.60 24821328

[131] Mazidi M de Caravatto PP Speakman JR Cohen RV. Mechanisms of action of surgical interventions on weight-related diseases: the potential role of bile acids. Obes Surg (2017) 27:826–36. doi: 10.1007/s11695-017-2549-1 28091894

[132] Barrasa JI Olmo N Lizarbe MA Turnay J. Bile acids in the colon, from healthy to cytotoxic molecules. Toxicol In Vitro (2013) 27:964–77. doi: 10.1016/j.tiv.2012.12.020 23274766

[133] Schaap FG Trauner M Jansen PLM. Bile acid receptors as targets for drug development. Nat Rev Gastroenterol Hepatol (2013) 11:55–67. doi: 10.1038/nrgastro.2013.151 23982684

[134] Watanabe M Houten SM Mataki C Christoffolete MA Kim BW Sato H. Bile acids 804-14. Nature (2006) 439(7075):484–9. doi: 10.1038/nature04330 16400329

[135] Garruti G De Fazio M Capuano P Martinez G Rotelli MT Puglisi F. Exercise and apulian hypocaloric diet affect adipokine changes and gastric banding-induced weight loss: a prospective study on severe obese subjects. Ann Med Surg. doi: 10.1016/j.amsu.2020.02.005 PMC7052402 32153773

[136] Rafferty EP Wylie AR Hand KH Elliott CE Grieve DJ Green BD. Investigating the effects of physiological bile acids on GLP-1 secretion and glucose tolerance in normal and GLP1R(-/-) mice. Biol Chem (2011) 392:539–46. doi: 10.1515/bc.2011.050 21521075

[137] Culnan DM Albaugh V Sun M Lynch CJ Lang CH Cooney RN. Ileal interposition improves glucose tolerance and insulin sensitivity in the obese zucker rat. Am J Physiol Gastrointest Liver Physiol (2010) 299:G751–G7. doi: 10.1152/ajpgi.00525.2009 PMC2950685 20634437

[138] Kohli R Kirby M Setchell KD Jha P Klustaitis K Woollett LA. Intestinal adaptation after ileal interposition surgery increases bile acid recycling and protects against obesityrelated comorbidities. Am J Physiol Gastrointest Liver Physiol (2010) 299:G652–60. doi: 10.1152/ajpgi.00221.2010 PMC2950688 20595624

[139] Mansuy-Aubert V Gautron L Lee S Bookout AL Kusminski C Sun K. Loss of the liver x receptor LXRalpha/beta in peripheral sensory neurons modifies energy expenditure. Elife (2015) 4:e06667. doi: 10.7554/eLife.06667 26076474 PMC4467361

[140] Kashihara H Shimada M Kurita N Sato H Yoshikawa K Higashijima J. Duodenaljejunal bypass improves diabetes and liver steatosis via enhanced glucagon-like peptide-1 elicited by bile acids. J Gastroenterol Hepatol (2015) 30:308–15. doi: 10.1111/jgh.12690 25088988

[141] Brighton CA Rievaj J Kuhre RE Glass LL Schoonjans K Holst JJ. Bile acids trigger GLP-1 release predominantly by accessing basolaterally located g protein-coupled bile acid receptors. Endocrinology (2015) 156:3961–70. doi: 10.1210/en.2015-1321 PMC4606749 26280129

[142] Pols TW Noriega LG Nomura M Auwerx J Schoonjans K. The bile acid membrane receptor TGR5: a valuable metabolic target. Dig Dis (2011) 29:37–44. doi: 10.1016/j.jhep.2010.12.004 21691102 PMC3128138

[143] Thomas C Gioiello A Noriega L Strehle A Oury J Rizzo G. TGR5-mediated bile acid sensing controls glucose homeostasis. Cell Metab (2009) 10:167–77. doi: 10.1016/j.cmet.2009.08.001 PMC2739652 19723493

[144] Kumar S Lau R Hall C Palaia T Brathwaite CE Ragolia L. Bile acid elevation after rouxen-y gastric bypass is associated with cardio-protective effect in zucker diabetic fatty rats. Int J Surg (2015) 24:70–4. doi: 10.1016/j.ijsu.2015.11.010 26563489

[145] Osto E Doytcheva P Corteville C Bueter M Dorig C Stivala S. Rapid and body weight-independent improvement of endothelial and high-density lipoprotein function after roux-en-Y gastric bypass: role of glucagon like peptide-1. Circulation (2015) 131:871–8. doi: 10.1161/CIRCULATIONAHA.114.011791 25673670

[146] Jahansouz C Xu H Hertzel AV Serrot FJ Kvalheim N Cole A. Bile acids increase independently from hypocaloric restriction after bariatric surgery. Ann Surg (2016) 264:1022–8. doi: 10.1097/SLA.0000000000001552 26655924

[147] Albaugh VL Flynn CR Cai S Xiao Y Tamboli RA Abumrad NN. Early increases in bile acids post roux-en-Y gastric bypass are driven by insulin-sensitizing, secondary bile acids. J Clin Endocrinol Metab (2015) 100:E1225–33. doi: 10.1210/jc.2015-2467 PMC4570157 26196952

[148] Albaugh VL Banan B Ajouz H Abumrad NN Flynn CR. Bile acids and bariatric surgery. Mol Aspects Med (2017) 56:75–89. doi: 10.1016/j.mam.2017.04.001 28390813 PMC5603298

[149] Ballesteros-Pomar MD Calleja S Diez-Rodriguez R Calleja-Fernandez A Vidal-Casariego A Nunez-Alonso A. Inflammatory status is different in relationship to insulin resistance in severely obese people and changes after bariatric surgery or diet-induced weight loss. Exp Clin Endocrinol Diabetes (2014) 122:592–6. doi: 10.1055/s-0034-1382035 25003361

[150] Liu N Zhao J Wang J Teng H Fu Y Yuan H. Farnesoid x receptor ligand CDCA suppresses human prostate cancer cells growth by inhibiting lipid metabolism *via* targeting sterol response element binding protein 1. Am J Transl Res (2016) 8:5118–24. PMC5126355 27904713

[151] Holst JJ. The physiology of glucagon-like peptide 1. Physiol Rev (2007) 87:1409–39. doi: 10.1152/physrev.00034.2006 17928588

[152] Yu H Ni Y Bao Y Zhang P Zhao A Chen T. Chenodeoxycholic acid as a potential prognostic marker for roux-en-Y gastric bypass in chinese obese patients. J Clin Endocrinol Metab (2015) 100:4222–30. doi: 10.1210/jc.2015-2884 26425885

[153] Shen H Zhang Y Ding H Wang X Chen L Jiang H. Farnesoid x receptor induces GLUT4 expression through FXR response element in the GLUT4 promoter. Cell Physiol Biochem (2008) 22:001–14. doi: 10.1159/000149779 18769028

[154] Ryan KK Tremaroli V Clemmensen C Kovatcheva-Datchary P Myronovych A Karns R. FXR is a molecular target for the effects of vertical sleeve gastrectomy. Nature (2014) 509:183–8. doi: 10.1038/nature13135 PMC4016120 24670636

[155] Düfer M Hörth K Wagner R Schittenhelm B Prowald S Wagner TF. Bile acids acutely stimulate insulin secretion of mouse β-cells *via* farnesoid x receptor activation and KATP channel inhibition. Diabetes (2012) 61:1479–89. doi: 10.2337/db11-0815 PMC3357280 22492528

[156] Kohli R Bradley D Setchell KD Eagon JC Abumrad N Klein S. Weight loss induced by roux-en-Y gastric bypass but not laparoscopic adjustable gastric banding increases circulating bile acids. J Clin Endocrinol Metab (2013) 98:E708–12. doi: 10.1210/jc.2012-3736 PMC3615197 23457410

[157] Steinert RE Peterli R Keller S Meyer-Gerspach AC Drewe J Peters T. Bile acids and gut peptide secretion after bariatric surgery: a 1-year prospective randomized pilot trial. Obes (Silver Spring) (2013) 21:E660–8. doi: 10.1002/oby.20522 23804517

[158] Koopmans HS Sclafani A Fichtner C Aravich PF. The effects of ileal transposition on food intake and body weight loss in VMH-obese rats. Am J Clin Nutr (1982) 35:284–93. doi: 10.1093/ajcn/35.2.284 7064889

[159] Strader AD Vahl TP Jandacek RJ Woods SC D'Alessio DA Seeley RJ. Weight loss through ileal transposition is accompanied by increased ileal hormone secretion and synthesis in rats. Am J Physiology-Endocrinology And Metab (2005) 288:E447–E53. doi: 10.1152/ajpendo.00153.2004 15454396

[160] Strader AD. Ileal transposition provides insight into the effectiveness of gastric bypass surgery. Physiol Behav (2006) 88:277–82. doi: 10.1016/j.physbeh.2006.05.034 16782138

[161] Strader AD Clausen TR Goodin SZ Wendt D. Ileal interposition improves glucose tolerance in low dose streptozotocin-treated diabetic and euglycemic rats. Obes Surg (2009) 19:96–104. doi: 10.1007/s11695-008-9754-x 18989728

[162] Patriti A Facchiano E Annetti C Aisa MC Galli F Fanelli C. Early improvement of glucose tolerance after ileal transposition in a non-obese type 2 diabetes rat model. Obes Surg (2005) 15:1258–64. doi: 10.1381/096089205774512573 16259883

[163] Tinoco A El-Kadre L Aquiar L Tinoco R Savassi-Rocha P. Short-term and mid-term control of type 2 diabetes mellitus by laparoscopic sleeve gastrectomy with ileal interposition. World J Surg (2011) 35:2238–44. doi: 10.1007/s00268-011-1188-2 21744166

[164] Ono M Shimizugawa T Shimamura M Yoshida K Noji-Sakikawa C Ando Y. Protein region important for regulation of lipid metabolism in angiopoietin-like 3 (ANGPTL3): ANGPTL3 is cleaved and activated in vivo. J Biol Chem (2003) 278(43):41804–41809. doi: 10.1074/jbc.M302861200 12909640

[165] Kaplan R Zhang T Hernandez M Gan FX Wright SD Waters MG. Regulation of the angiopoietin-like protein 3 gene by LXR. J Lipid Res (2003) 44(1):136–43. doi: 10.1194/jlr.M200367-JLR200 12518032

[166] Shimamura M Matsuda M Ando Y Koishi R Yasumo H Furukawa H. Leptin and insulin down-regulate angiopoietin-like protein 3, a plasma triglyceride-increasing factor. Biochem Biophys Res Commun (2004) 322(3):1080–5. doi: 10.1016/j.bbrc.2004.08.024 15336575

[167] Inukai K Nakashima Y Watanabe M Kurihara S Awata T Katagiri H. ANGPTL3 is increased in both insulin-deficient and -resistant diabetic states. Biochem Biophys Res Commun (2004) 317(4):1075–1079. doi: 10.1016/j.bbrc.2004.03.151 15094378

[168] Chi X Britt EC Shows HW Hjelmaas AJ Shetty SK Cushing EM. ANGPTL8 promotes the ability of ANGPTL3 to bind and inhibit lipoprotein lipase. Mol Metab (2017) 6(10):1137 –1149. doi: 10.1016/j.molmet.2017.06.014 PMC5641604 29031715

[169] Haller JF Mintah IJ Shihanian LM Stevis P Buckler D Alexa-Braun CA. ANGPTL8 requires ANGPTL3 to inhibit lipoprotein lipase and plasma triglyceride clearance. J Lipid Res (2017) 58(6):1166–1173. doi: 10.1194/jlr.M075689 28413163 PMC5454515

[170] Kitazawa M Ohizumi Y Oike Y Hishinuma T Hashimoto S. Angiopoietin-related growth factor suppresses gluconeogenesis through the akt/forkhead box class O1-dependent pathway in hepatocytes. J Pharmacol Exp Ther (2007) 323(3):787e793. doi: 10.1124/jpet.107.127530 17804676

[171] Kadomatsu T Tabata M Oike Y. Angiopoietin-like proteins: emerging targets for treatment of obesity and related metabolic diseases. FEBS J (2011) 278(4):559–64. doi: 10.1111/j.1742-4658.2010.07979.x 21182596

[172] Xu A Lam MC Chan KW Wang Y Zhang J Hoo RL. Angiopoietin-like protein 4 decreases blood glucose and improves glucose tolerance but induces hyperlipidemia and hepatic steatosis in mice. Proc Natl Acad Sci U.S.A. (2005) 102(17):6086–91. doi: 10.1073/pnas.0408452102 PMC1087912 15837923

[173] Abu-Farha M Al-Khairi I Cherian P Chandy B Sriraman D Alhubail A. Increased ANGPTL3, 4 and ANGPTL8/betatrophin expression levels in obesity and T2D. Lipids Health Dis (2016) 15(1):181. doi: 10.1186/s12944-016-0337-x 27733177 PMC5062897

[174] Cushing EM CHIX Sylvers KL Shetty SK Potthof MJ Davies BSJ. Angiopoietin-like 4 directs uptake of dietary fat away from adipose tduring fasting. Mol Metab (2017) 6(8):809–18. doi: 10.1016/j.molmet.2017.06.007 PMC5518724 28752045

[175] Nishimura T Nakatake Y Konishi M Itoh N. Identification of a novel FGF, FGF-21, preferentially expressed in the liver. Biochim Biophys Acta (2000) 1492(1):203–6. doi: 10.1016/S0167-4781(00)00067-1 10858549

[176] Hotta Y Nakamura H Konishi M Murata Y Takagi H Matsumura S. Fibroblast growth factor 21 regulates lipolysis in white adipose tissue but is not required for ketogenesis and triglyceride clearance in liver. Endocrinol. (2009) 150(10):4625–33. doi: 10.1210/en.2009-0119 19589869

[177] Bon Durant LD Ameka M Naber MC Markan KR Idiga SO Acevedo MR. FGF21 regulates metabolism through adipose-dependent and -independent mechanisms. Cell Metab (2017) 25(4):935–944. 28380381 10.1016/j.cmet.2017.03.005 PMC5494834

[178] Dang F Wu R Wang P Wu Y Azam MS Xu Q. Fasting and feeding signals control the oscillatory expression of Angptl8 to modulate lipid metabolism. Sci Rep (2016) 6:36926. doi: 10.1038/srep36926 27845381 PMC5109406

[179] Yang L Yin R Wang Z Wang X Zhang Y Zhao D. Circulating Angptl3 and Angptl8 are increased in patients with hypothyroidism. BioMed Res Int (2019), 3814687. doi: 10.1155/2019/3814687 31380419 PMC6662479

[180] Grzyb J Latowski D Strzałka K. Lipocalins - a family portrait. J Plant Physiol (2006) 163(9):895 –915. doi: 10.1016/j.jplph.2005.12.007 16504339

[181] Willis SA Sargeant JA Yates T Takamura T Takayama H Gupta V. Acute hyperenergetic, high-fat feeding increases circulating FGF21, LECT2, and fetuin-a in healthy men. J Nutr (2020) 150(5):1076 –85. doi: 10.1093/jn/nxz333 31919514

